# IL-10 producing B cells rescue mouse fetuses from inflammation-driven fetal death and are able to modulate T cell immune responses

**DOI:** 10.1038/s41598-019-45860-2

**Published:** 2019-06-27

**Authors:** Mandy Busse, Kim-Norina Jutta Campe, Desiree Nowak, Anne Schumacher, Susanne Plenagl, Stefanie Langwisch, Gisa Tiegs, Annegret Reinhold, Ana Claudia Zenclussen

**Affiliations:** 1 Experimental Obstetrics and Gynecology, Medical Faculty, Otto-von-Guericke University, Magdeburg, Germany; 2 Institute of Experimental Immunology and Hepatology, University Medical Center, Hamburg-Eppendorf, Hamburg Germany; 3 Institute for Molecular and Clinical Immunology, Medical Faculty, Otto-von-Guericke University, Magdeburg, Germany

**Keywords:** Pathogenesis, Experimental models of disease, Translational immunology

## Abstract

Understanding the mechanisms leading to fetal death following maternal subclinical infections is crucial to develop new therapeutic strategies. Here we addressed the relevance of IL-10 secreting B cells (B10) in the maintenance of the immune balance during gestation. µMT females lacking mature B cells presented normal pregnancies, although their fetuses were smaller and their Treg pool did not expand as in B cell sufficient controls. Pregnant µMT females were more susceptible to LPS despite having less Treg; their fetuses died at doses compatible with pregnancy in WT animals. Adoptive transfer of IL-10 negative B effector cells or B cells from IL-10 deficient mice did not modify this outcome. The transfer of B10 cells or application of recombinant murine IL-10 reduced the fetal loss, associated with a normalization of Treg numbers and cytokine modulation at the feto-maternal interface. B cell-derived IL-10 suppressed the production of IL-17A and IL-6 by T cells and promoted the conversion of naïve cells into Treg. B10 cells are required to restore the immune balance at the feto-maternal interface when perturbed by inflammatory signals. Our data position B cells in a central role in the maintenance of the balance between immunity and tolerance during pregnancy.

## Introduction

Pregnancy success depends upon complex multicellular interactions that enable the establishment of immunological tolerance towards the fetus while maintaining immunity against pathogens. Subclinical infections that jeopardize the so important immune equilibrium by inclining the balance to the inflammatory side are a major problem faced daily by gynecologists. In particular intrauterine infections are associated with fetal morbidity and mortality^1^. As a general mechanism against infections, maternal immune cells secrete inflammatory cytokines^2,3^, among which TNF-α and IFN-γ are relevant^4–6^. An exaggerated influx of Th1 cytokines to combat infections can, however, kill the progeny^7^. IL-10 is the most potent anti-inflammatory cytokine able to counteract the negative effects of pro-inflammatory cytokines, in particular TNF-α. IL-10 is not required for pregnancy under normal conditions as revealed by the use of IL-10 knockout mice^8^ but it turns out to be indispensable under infection conditions as elegantly shown in LPS models^9,10^. Moreover, the application of exogenous IL-10 can rescue from LPS-induced fetal loss^9,10^ and from spontaneous fetal loss^10,11^. It was long assumed that the main source of IL-10 was the placenta itself^12^. However, innate and adaptive immune cells are able to secrete IL-10 as well, albeit in lower concentrations. We have recently shown that IL-10 producing CD19^+^CD24^hi^CD27^+^ regulatory B cells^13^ are increased in number during human pregnancy^14^. Moreover, IL-10 producing B cells (B10 cells) from healthy pregnant women could suppress TNF-α production by T cells^14^. In mice, B10 cells expand during pregnancy as well as we recently reported^15^. Interestingly, it seems that the placenta itself and in particular its product human chorionic gonadotropin (hCG) can induce IL-10 production by B cells^16^. The absence, diminution or dysregulation of regulatory B cells or B10 cells^17–19^ have recently shown to be relevant for a variety of pathologies, including systemic lupus erythematosus, multiple sclerosis, rheumatoid arthritis and chronic lymphocytic leukemia^20–22^. The possible participation of IL-10 producing B cells in the prevention of fetal damage or death after infections has not been explored in deep. Here, we characterized pregnancy outcome in mice devoid of mature B cells, namely µMT mice, that carry a disruption of the membrane exon of the immunoglobulin µ-chain gene^23^. We also studied the effect of LPS, that mimics a gram-negative infection^6^, on the pregnancy outcome of these mice. For this, we first titrated LPS in gd10 μMT and wild type mice and then chose a concentration that causes no harm in wild type animals but provokes fetal death in µMT mice.

Our study shows that B cell-secreted IL-10 is required if pregnancy is jeopardized by the presence of LPS. B cell contribution can obviously not be compensated by expansion or activation of another cell population. Thus, these cells emerge as essential to keep the cell and cytokine balance needed at the feto-maternal interface. Hence, we propose IL-10 secreting B cells as important regulators of immune balance during pregnancy.

## Results

### Pregnancy in B cell deficient µMT mice resulted in smaller embryo sizes

**Figure 1:**
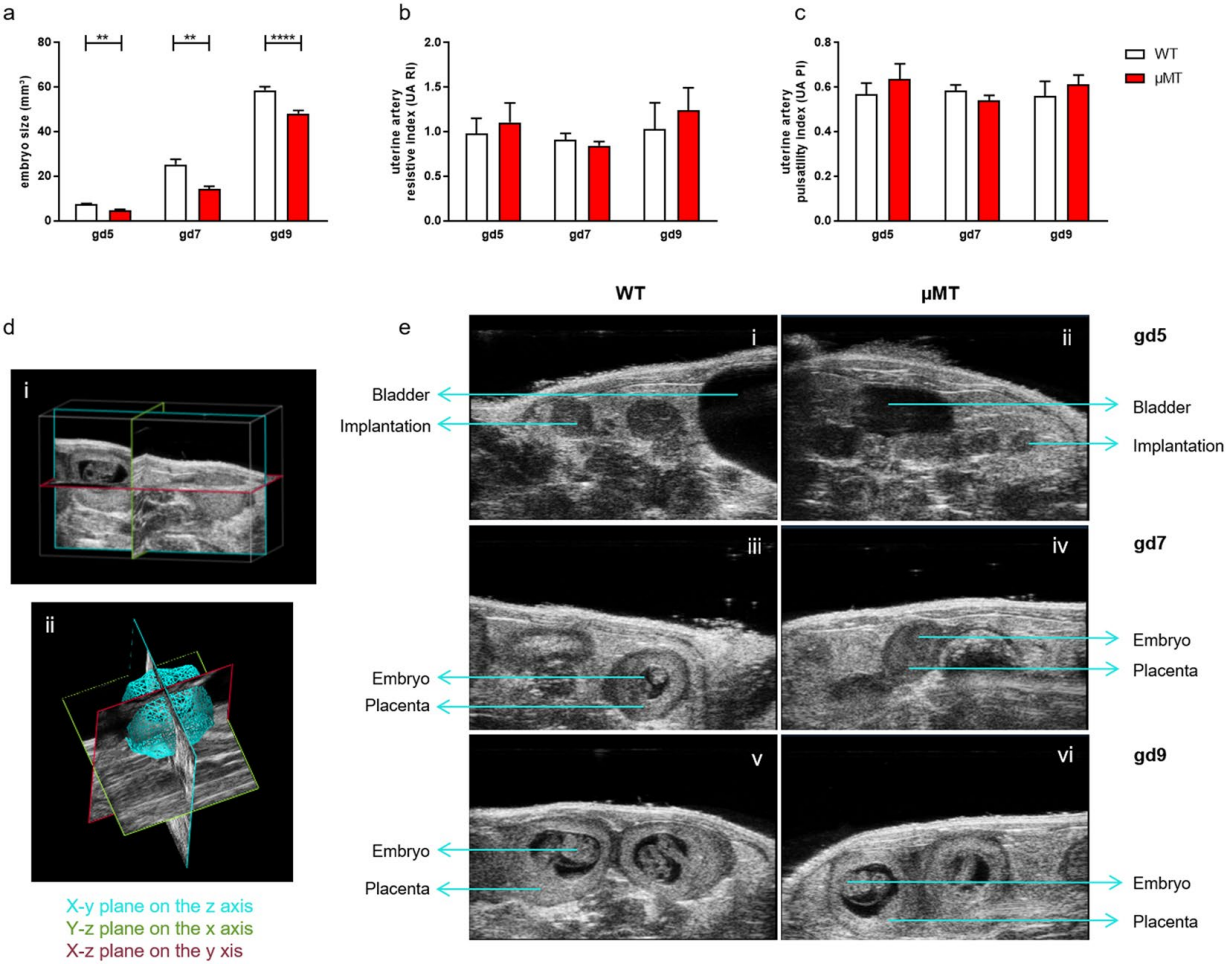
Implantation sizes of µMT mice were smaller than those of wild type (WT) controls at days 5, 7 and 9 of pregnancy. Implantation sizes (**a**), artery pulsatility index (UA PI) (**b**) and the uterine artery resistive index (UA RI, (**c**) were measured by high frequency ultrasound (VEVO 2100) at gestation day (gd) 5, gd7 and gd9. (**d**) Shows how the 3D measurement is performed. (**d**) (i) depicts three single, slidable image slice views presented on the x, y and z planes. Each plane presents its color outline, with blue showing the x-y plane on the z axis, green the y-z plane on the x axis and red the x-z plane on the y axis. In (**d**) (ii) a three dimensional view of the acquired data can be observed. (**e**) depicts representative pictures from WT controls (i, iii, v) and µMT implantations (ii, iv and vi) at gd 5 (i, ii), 7 (iii, iv) and 9 (v and vi) respectively. The data obtained after imagining was analyzed using the Kruskal-Wallis test and Mann-Whitney-*U* test; data are shown as mean ± SEM; n = 4–6 dams/group; n = 1–3 fetuses/dam; **p < 0.01; ****p < 0.0001.

BALB/c-mated µMT females had decreased implantation numbers (mean 7.4) compared to WT mice (mean 8.4; p = 0.0195). Embryo size, as measured by high frequency ultrasound at gd5, gd7 and gd9 was significantly reduced compared to WT embryos (Fig. 1a; 3D analysis shown in Fig. 1d; exemplary pictures of fetuses at gd 5, 7 and 9 are shown in Fig. 1e). There were no alterations in the velocity of the uterine artery as shown by the resistive index (RI) and the pulsatility index (PI) values at gd5, gd7 and gd9 (Fig. 1b,c). Other than being smaller, the histology of µMT implantations was comparable to the controls (Supplementary Fig. 1) and the physiologically relevant allogeneic mating did not show intrauterine fetal death.

### Naïve µMT mice presented a normal Treg pool; however the lack of mature B cells in these mice correlated with their inability to expand the Treg pool upon pregnancy as WT mice normally do

**Figure 2:**
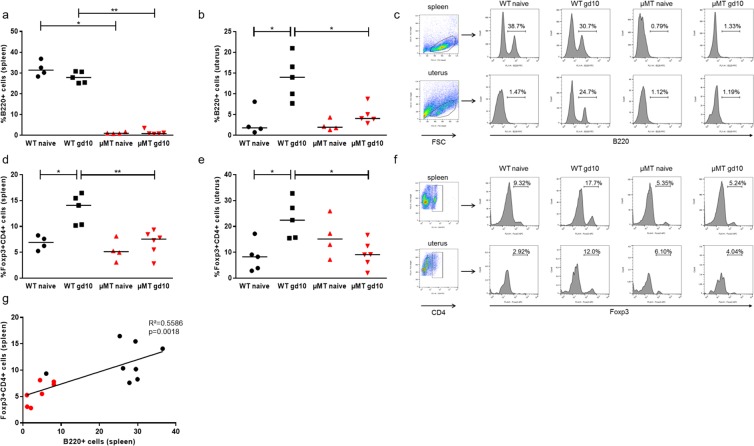
B cell deficient µMT mice failed to expand the pool of splenic and uterine Treg cells as wild type (WT) controls did. (**a**) The number of B220+ splenic B cells remained stable in WT mice at midgestation compared to naïve mice. (**b**) In uterine tissue, the number of B cells increased in WT mice that were pregnant at gd10 when compared to naïve WT animals. In µMT mice, the frequency of B cells was, as expected, almost undetectable and this did not change upon pregnancy neither in spleen nor in uterus. Representative plots are shown in (**c**). (**d**,**e**) The number of regulatory T cells (Treg) was increased in pregnant WT mice at gd10 in spleen (**c**) and uterus (**d**) when compared to non-pregnant control females, while the Treg levels remained unaltered in pregnant µMT mice when compared to non-pregnant µMT mice (**d**,**e**). (**f**) Shows representative plots. (**g**) The number of splenic Treg cells correlated with the number of B220+ B cells in both WT and µMT mice. Data are analyzed using Kruskal-Wallis test and Mann-Whitney test and shown as median. n = 4–6 mice/group; *p < 0.05; **p < 0.01.

Flow cytometry analysis of B220, CD19, IgM and IgD confirmed that µMT mice lack mature B cells in spleen (Fig. 2a, dot plots in Supplementary Fig. 1a). The same was true for blood, peritoneal lavage and lymph nodes (data not shown). In uterus, a small proportion of B220 positive cells could be detected in µMT mice (Fig. 2b, Supplementary Fig. 2b). In WT mice, pregnancy did not change the total B cell pool in the periphery (Fig. 2a) but provoked an increase in the number of total B cells (B220+ cells) in uterus at gd10 compared to non-pregnant females (p = 0.0317, Fig. 2b, Supplementary Fig. 2b) that was not registered in µMT mice (Fig. 2b,c). As expected^24^, pregnancy (gd10) expanded the pool of Foxp3+ Treg cells of WT mice in spleen (p = 0.0159, Fig. 2d) and uterus (p = 0.0317, Fig. 2e,f and Supplementary Fig. 2c,d). This pregnancy-induced Treg expansion was not observed in µMT mice that had significantly decreased Treg numbers at gd10 in both spleen (Fig. 2d, p = 0.0043) and uterus (p = 0.0173; Fig. 2e; representative plots Fig. 2f) when compared to the pregnant controls. This further correlated with the numbers of B cells (Fig. 2g).

### Despite non-expanded Treg levels, pregnant µMT mice exhibited an increased susceptibility to LPS that provoked intrauterine fetal death

**Figure 3:**
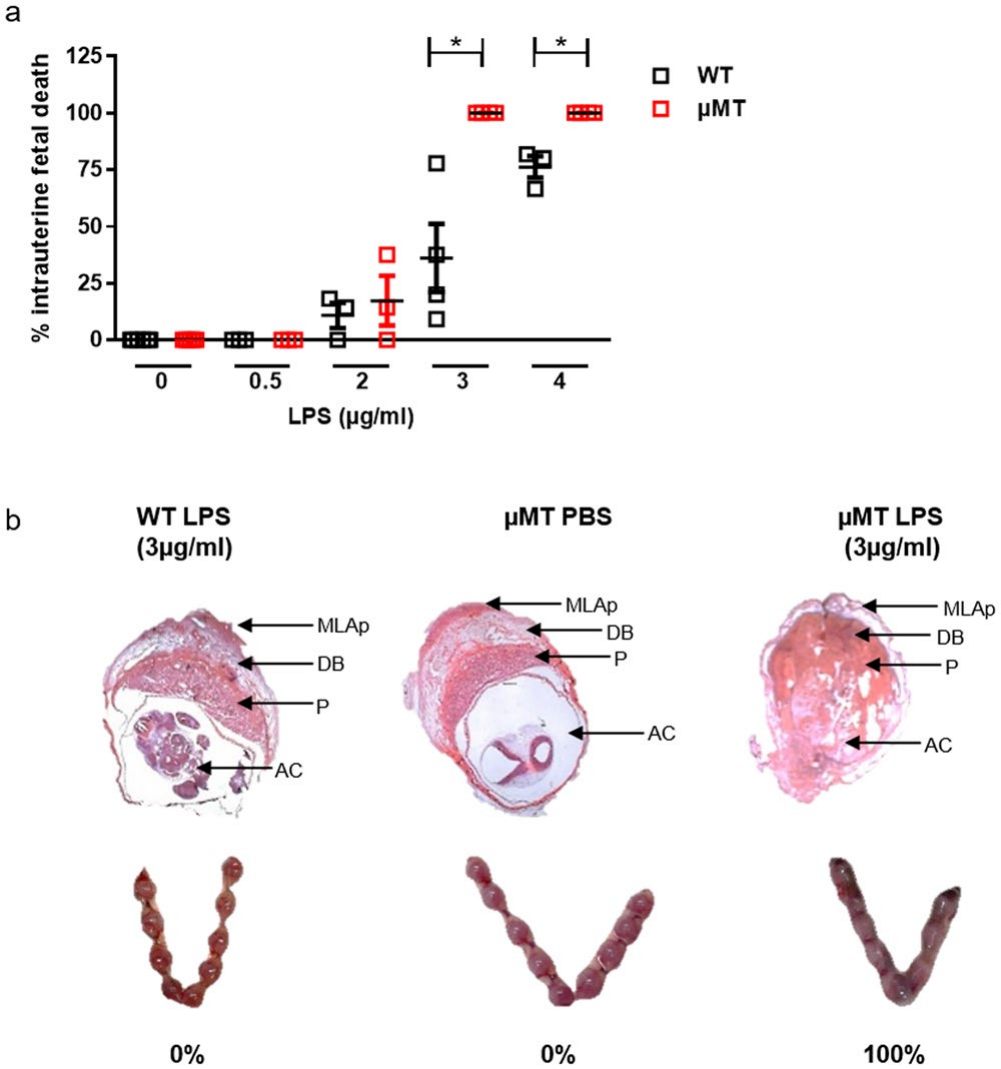
Pregnant µMT mice had increased susceptibility to LPS and present intrauterine fetal death (IUFD) at doses that caused no harm in control wild type (WT) mice. (**a**) Intraperitoneal (i.p.) injection of 0.5 and 2 µg/ml LPS had similar effects on WT and µMT pregnant mice. 3 µg/ml and 4 µg/ml LPS induced 100% IUFD in µMT mice that were pregnant at gd10, while WT mice had significantly less dead fetuses after LPS treatment. (**b**) Shows representative pictures of H&E stained whole implantation sites (WIS; upper part); arrows indicate AC = amniotic cavity containing the fetus; P = placenta; DB = decidua basalis; MLAp = mesometrial lymphoid aggregate of pregnancy). H&E staining of WIS performed 24 h after LPS application illustrated that fetuses in µMT mice were already degraded compared to intact fetuses in WT females. In the lower part, pictures of uteri of WT and µMT mice 24 h after LPS or PBS treatment indicate that even at the naked eye the dead fetuses can be observed in the µMT group. Data were analyzed by Kruskal-Wallis test and Mann-Whitney test; n = 3–8 mice/group for the titration; *p < 0.05.

To investigate whether the lack of mature B cells affects the susceptibility to LPS-induced intrauterine fetal death (IUFD), we injected 0.5, 2, 3 or 4 µg/ml LPS i.p. to WT and µMT mice at gd10 (midpregnancy) and determined the rate of fetal death 24 h later (Fig. 3a). Similar outcomes were observed in all groups when employing 0.5 or 2 µg/ml LPS. At 3 µg/ml LPS, all fetuses died in the in µMT group, while only one third did in the WT group (p = 0.0265). 4 µg/ml LPS increased the IUFD rate in WT mice to 76%, compared to 100% fetal death in µMT mice (p = 0.0436). At 10 µg/ml both groups presented 100% IUFD (data not showed). 3 µg/ml LPS was the chosen concentration for the forthcoming experiments since it was the lowest concentration inducing significant differences between WT and µMT mice. Representative pictures of uteri obtained from LPS-treated µMT and WT mice and PBS-injected control µMT mice are shown in Fig. 3b. H&E staining of whole implantation sites (WIS) 24 h after LPS illustrated that fetuses in µMT mice were already degraded compared to intact fetuses in WT.

### Administration of IL-10-producing B cells or recombinant murine IL-10 rescued from LPS-induced intrauterine fetal death, while the transfer of IL-10 negative B effector cells or total B cells from IL-10 deficient mice did not

**Figure 4:**
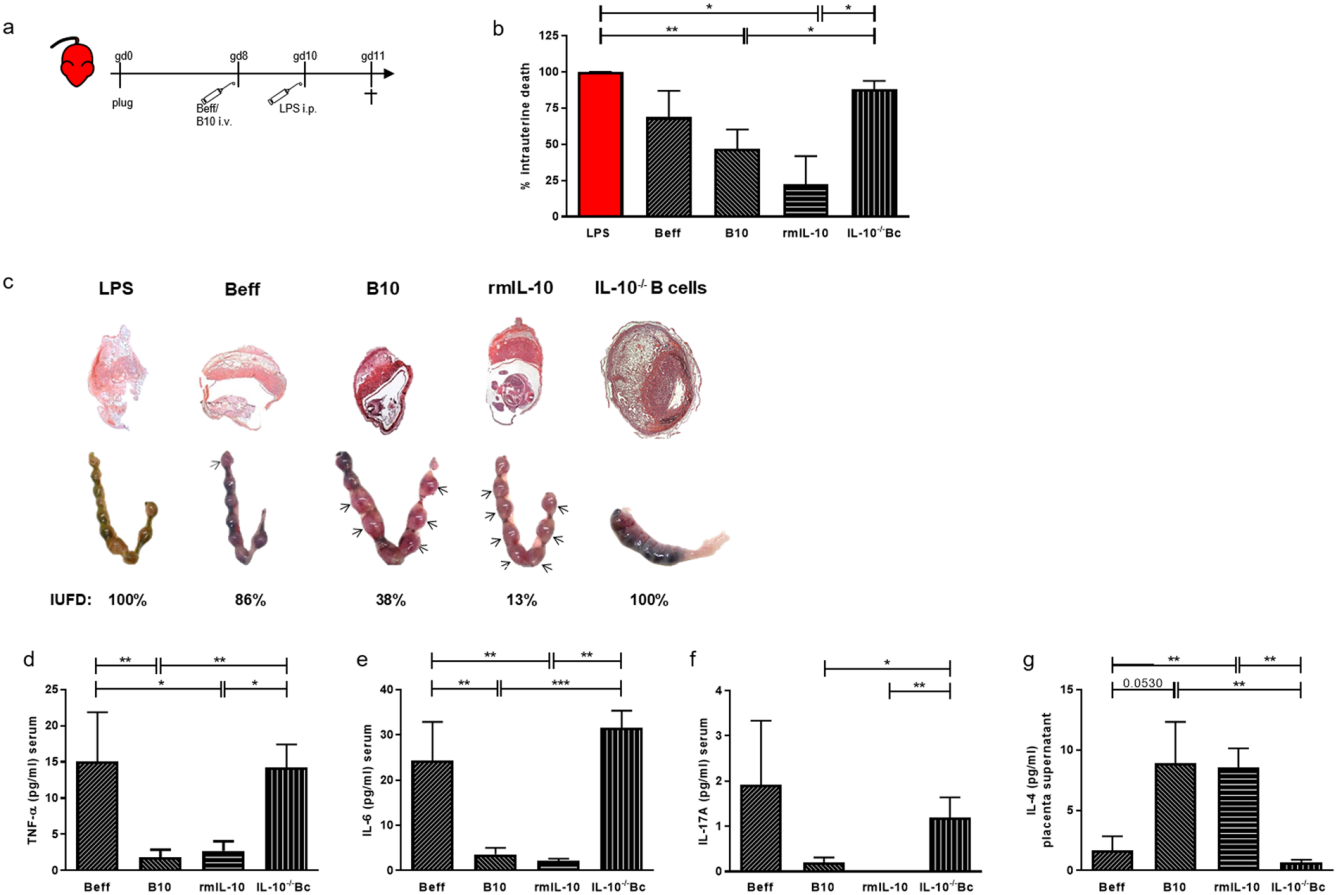
The transfer of IL-10-producing B cells or rmIL-10 treament protected against intrauterine fetal death while the transfer of effector B cells or total B cells from IL-10^−/−^ mice did not. (**a**) Depicts the experimental design of the adoptive cell transfer experiment. µMT mice were supplemented i.v. with Beff or B10 cells obtained from WT mice or total B cells obtained from IL-10-deficient mice (IL-10^−/−^ Bc) at gd8, 2d before LPS challenge at gd10. Another group received 2.5 µg rmIL-10 2d before LPS challenge. 24 h after LPS, the mice were sacrificed. (**b**) Shows the IUFD for all groups. Administration of B10 cells or rmIL-10 reduced the IUFD rate, the transfer of Beff or IL-10^−/−^ B cells had no significant effect on the IUFD compared to controls. (**c**) Shows representative pictures of H/E stained implantations (upper part) and photos showing uteri of µMT mice (lower part) supplemented with Beff, B10 cells or IL-10^−/−^ B cells or injected with rmIL-10 at gd8, followed by LPS at gd10. Uteri of treated mice were obtained 24 h after LPS. Number of implantations was counted and the number of living/dead implantations was calculated. The % of IUFD showed here corresponds to the examples depicted in (**d**). Arrows indicate living fetuses. Injection of B10 cells or rmIL-10 reduced TNF-α (**d**), IL-6 (**e**) and IL-17 (**f**) concentrations (in pg/ml) and increased IL-4 in the placenta supernatant (**g**) compared to Beff− or IL-10^−/−^ B cell-treated groups. Kruskal-Wallis test and Mann-Whitney-*U* test were used to analyze the data. N = 5–8 mice/group; *p < 0.05, **p < 0.01, ***p < 0.001.

To understand whether LPS-driven fetal death in µMT mice owed to the lack of B cells, we next administered different B cell populations prior to the 3 µg/ml LPS challenge. 2d prior to LPS challenge at gd10 µMT, females were reconstituted i.v. with either IL-10-negative B effector cells (Beff) or IL-10-producing B10 cells (Fig. 4a, Supplementary Fig. 3). The adoptive transfer of Beff cells did not significantly reduce the IUFD rate (p = 0.0606; Fig. 4b), but the transfer of B10 cells into µMT mice effectively reduced IUFD (p = 0.0064; Fig. 4b), suggesting that B cell-secreted IL-10 is the decisive factor in preventing fetal death after LPS challenge. To confirm this, two different sets of experiments were performed. Firstly, B cells from IL-10-deficient (IL-10^−/−^) mice were adoptively transferred into gd8 pregnant µMT mice. Fetal death following LPS was comparable to LPS challenge alone, thus significantly enhanced compared to the reconstitution with B10 cells (p = 0.0064), confirming IL-10 secretion as the factor conferring protective abilities to B10 cells. Secondly, µMT mice were injected with 2.5 µg rmIL-10 at gd8, followed by LPS challenge at gd10. rmIL-10 significantly reduced IUFD (p = 0.0281), underlining the relevance of this cytokine in our animal model (Fig. 4b). Representative pictures of WIS and uteri of treated mice are shown in Fig. 4c. We next concentrated on the cytokine levels of cell-transferred LPS-treated animals. Interestingly, pregnant females transferred with B10 cells or treated with rmIL-10 had decreased levels of TNF-α (Fig. 4d), IL-6 (Fig. 4e) and IL-17 (Fig. 4f) in serum compared to animals that received Beff or IL-10^−/−^ B cells. IL-4 levels were not affected in serum (data not shown). However, when determining the cytokine milieu at the feto-maternal interface, we observed that placenta explants from animals treated with B10 or rmIL-10 secreted elevated levels of IL-4 compared to animals treated with Beff cells or IL-10^−/−^ B cells (Fig. 4g). No differences were observed in supernatants of placenta explants regarding inflammatory cytokines (data not shown).

To determine whether the time point of B cell transfer matters, we carried out experiments in which the transfer of cells took place 2 h before LPS challenge at gd10 (see scheme Supplementary Fig. 4a). We observed the same positive effect of B10 cells vs Beff cells in diminishing the IUFD rate (Supplementary Fig. 4b), macroscopic appearance of WIS (Supplementary Fig. 4c), concentration of TNF-α, IL-6 and IL-17A in serum (Supplementary Fig. 4d–f), or of IL-4 in placenta supernatants (Supplementary Fig. 4g) compared to the injection of B10 cells 2 days before LPS challenge.

### Understanding the mechanisms underlying IL-10 protection: B10 cells from pregnant mice were able to modulate T cell phenotype and their cytokine secretion ability

**Figure 5:**
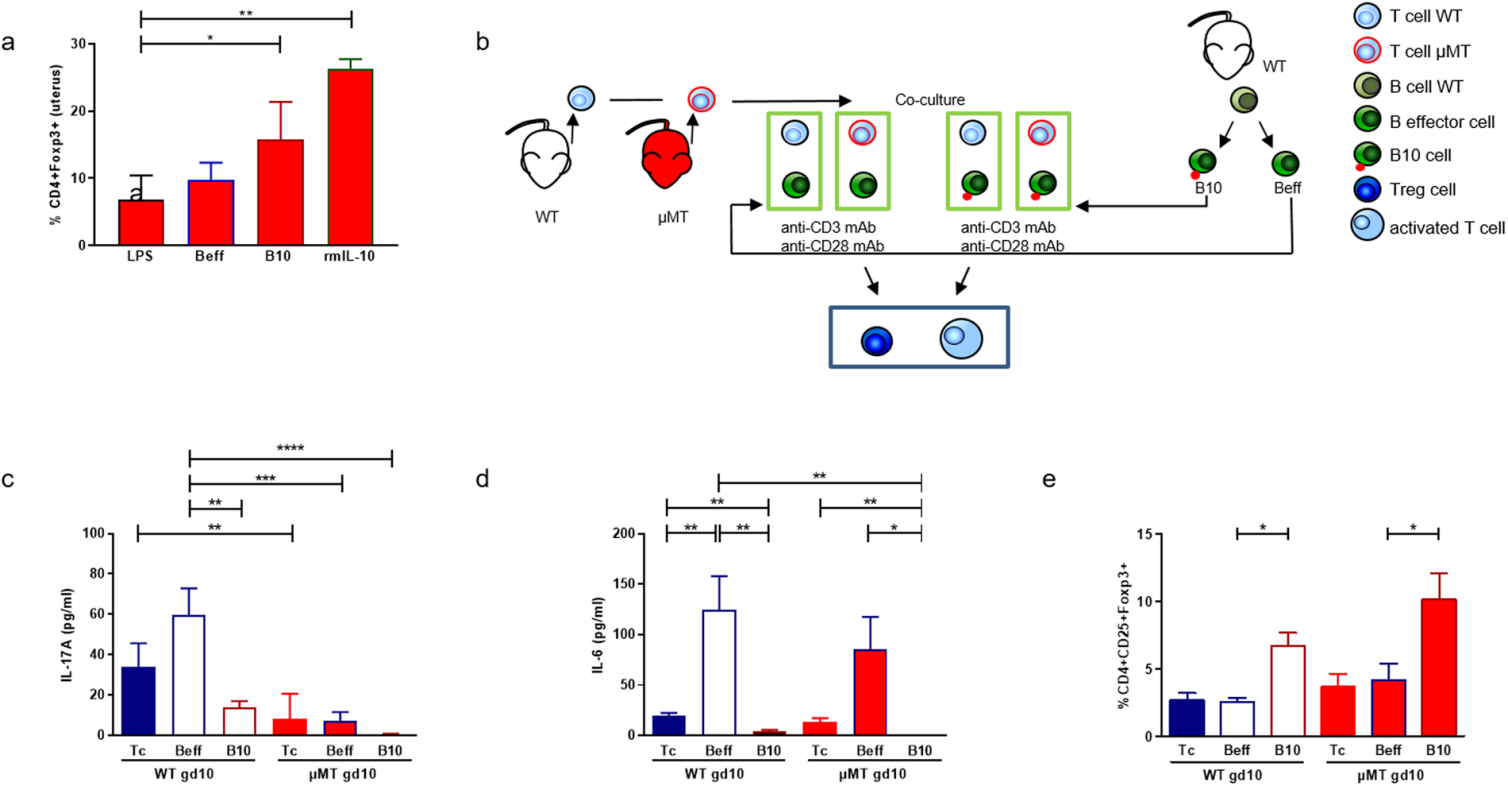
B10 cells influence T cell functionality. (**a**) Shows the frequency of CD4+Foxp3+ Treg cells in the uterus of µMT mice injected with Beff or B10 cells or treated with rmIL-10 at gd8, followed by LPS at gd10 or LPS alone and analyzed 24 h later. (**b**) Shows a scheme of the *in vitro* co-culture setting. Beff or B10 cells isolated from WT mice were co-cultured with naïve CD4+CD25− T cells, obtained from WT or µMT mice that were pregnant at gd10. After 72 h stimulation with anti-CD3/28, the secretion of IL-17A (**c**) and IL-6 (**d**) as well as their conversion into CD4+CD25+Foxp3+ Treg cells (**e**) were determined either by bead array cytometry (**c**,**d**) or flow cytometry (**e**) and further compared to the data obtained following the culture of T cells alone (Tc). Data was analyzed by repeated measures two-way ANOVA followed by Bonferroni correction for multiple analysis and the data are shown as mean ± SEM. N = 4–6 mice/group; *p < 0.05, **p < 0.01, ***p < 0.001, ****p < 0.0001.

The application of B10 cells or rmIL-10 at gd8, followed by LPS at gd10, significantly increased the number of uterine CD4+ Foxp3+ Treg cells (Fig. 5a) compared to animals only challenged with LPS. To mimic this situation, we performed *in vitro* co-culture assays to understand the influence of B cell-derived IL-10 on T cells in pregnancy (experimental set up in Fig. 5b). Beff cells or B10 cells were added to CD4+CD25− naïve T cells obtained from WT or µMT mice at gd10 that were cultured with anti-CD3/CD28 antibodies. Compared to T cells cultured alone (p = 0.0087) or with Beff cells (p = 0.0015), the addition of B10 cells reduced the secretion of IL-17A by WT T cells (Fig. 5c). µMT T cells had a similar effect; however, the IL-17A levels were much lower. T cells from gd10 WT mice produced more IL-17A when co-cultured with Beff (p = 0.0003) or B10 cells (p < 0.0001) than µMT T cells did (Fig. 5c). The secretion of IL-6 by T cells was diminished when B10 cells were added to WT T cells (p = 0.0063) or µMT T cells (p = 0.0493) compared to co-cultures between T and Beff cells or T cells alone. The same was true for µMT T cells cultured alone (p = 0.0022; Fig. 5d). Secretion of IL-6 by T cells was increased after the addition of Beff cells to WT T cells (p = 0.0022) as compared to T cells cultured alone.

Culturing CD4+CD25− naïve T cells from gd10 WT and µMT mice with Beff cells or alone did not affect the frequency of converted Treg cells (Fig. 5e). However, the addition of B10 cells to naïve CD4+CD25− T cells significantly augmented the proportion of CD4+CD25+Foxp3+ Tregs in cells of WT (p = 0.0069), but also of µMT mice (p = 0.0348; Fig. 5e).

## Discussion

Subclinical infections during pregnancy can have fatal consequences likewise pre-term birth or even intrauterine fetal death. Knowing that IL-10 is able to prevent LPS-induced fetal death in mice^10^ and taking into account that in several models, B cell-derived IL-10 can counteract inflammatory responses^19,25^, we explored whether IL-10 produced by B cells may have a beneficial effect in pregnancy by combating undesired inflammatory responses.

B cells are present at low numbers at the feto-maternal interface and they were long believed to have no relevance in pregnancy; however, recent data uncover that B10 cells may be important players in early pregnancy^14–16^. Thereby the question arises whether B cell depletion might cause complications for mother and unborn. For treating autoimmune disorders such as Rheumatoid Arthritis or Multiple Sclerosis, depletion of B cells was shown to have a good response rate. Rituximab is a human/murine chimeric IgG mAb targeting CD20 which can cross the placenta from gestational week 16 onwards^26^. Even though the majority of pregnancy reports state that the treatment is compatible with term live births, spontaneous abortions, preterm deliveries, low-for-gestational-age, intrauterine growth restriction and preeclamsia were associated with Rituximab use before or during pregnancy^27–30^. Moreover, this medication might increase the risk of immunosuppression and subsequent infection for the neonate, strongly indicating the need for further research on the consequences of B cell depletion in pregnancy.

Our data show that B cell numbers in pregnancy correlate with Treg cells and this is of particular relevance as maternal Treg cells are not only important for the establishment and maintenance of pregnancy^31,32^ but have been also suggested to dictate the susceptibility to prenatal and pregnancy complications (reviewed in^33^). Specifically, it is believed that the pregnancy-specific Treg expansion makes females more susceptible to infections. In naïve µMT mice, no changes in T cell or Treg cells were found compared to the controls. However, they fail to expand the existing Treg population upon pregnancy as wild type females do. A defect in the upregulation of the immunoregulatory cytokines IL-10 and TGF-β^34^ and in the induction of Treg cells^35^ was described upon oral immune tolerance induction in these mice. Here, a follow-up of gestational parameters by serial high frequency ultrasound measurements revealed a reduced litter size as well as a decreased size of the embryos in µMT mice compared to WT females reinforcing the meaning of B cells for a healthy pregnancy.

Natural tolerance to fetal antigens is a complex process involving finely regulated mechanisms. By using elegant models, Rowe and colleagues introduced the idea that one relevant mechanism for tolerance, namely maternal Treg expansion, creates holes in the host defense that confer increased susceptibility to infections during pregnancy^36^. However, we think that not only one cell type but rather a network of cells defines the balance between tolerance and immunity in pregnancy. Our assumption is supported by the data presented in this paper; µMT mice failed to expand their Treg population upon pregnancy but this did not confer them any protection against inflammation as it would be expected following Rowe’s theory. On the contrary, fetuses from mice devoid of mature B cells were more susceptible to LPS and they died intrauterine. These observations were done on gd10-gd11, namely at the end of the midpregnancy period, a time point we chose because of its translational value. Inflammation as for example following (subclinical) infections can lead to intrauterine fetal death in humans and this usually occurs at midpregnancy.

Normally, B cells are involved in the defense of pathogens by several mechanisms. They enhance innate immune responses^37^ which are required for the regulation of the local T cell activation as showed in a model of genital tract infection by *Chlamydia muridarum*^38^. Besides, TLR agonists like LPS are known to induce IL-10 competent “regulatory” B cells^39^ that limit the severity and damage caused by an inflammatory response among other mechanisms by affecting Treg number^40^.

To prove the hypothesis that B10 cells might improve fetal survival by restoring the balance that inflammatory signals may cause, LPS-treated B cells were separated according to their IL-10 secretion capacity into IL-10 negative B effector cells (Beff) and IL-10-producing (B10) cells and injected 2d before LPS challenge. We observed diminished levels of TNF-α and IL-6 in serum in B10-treated mice; both cytokines being induced in the Beff-treated mice. We performed experiments seeking to see whether B10 cells applied at 2 h vs 2d before LPS challenge had different outcomes, but this was not the case. Systemically, B10 cells limit the pro-inflammatory immune response, at least partially by favouring the differentiation of naïve T cells into Treg cells. Treg cells are effective inhibitors of Th1 and Th2 cell activation^41,42^ and obviously not always enablers of infection as proposed previously^33^. These data highlight the importance of a fine-tuned cell and cytokine balance that might be partly influenced by B cells.

Beff cells that do not secrete IL-10 or B cells isolated from IL-10^−/−^ animals could not revert the LPS-driven inflammation while B10 cells could. Thus, it is clear that µMT animals are not prone to LPS-induced fetal death because they lack mature B cells but because they lack B cells that can produce IL-10. As other cells are able to produce IL-10 systemically and at the feto-maternal interface, the second conclusion of our data is that B cell derived IL-10 is of crucial relevance and its contribution cannot longer be compensated if the immune balance is in danger because of inflammatory signals. IL-10^−/−^ mice present apparently normal pregnancies and the application of low doses of LPS provoked IUFD that can be reverted by the addition of rmIL-10^9,10^. To confirm that IL-10 is the major mechanism as to why B10 cells can attenuate LPS-induced IUFD, we treated µMT animals with rmIL-10 before LPS challenge. This resulted in a comparable outcome as the B10 cell transfer: reduced IUFD rate, decreased TNF-α, IL-6 and IL-17 and increased IL-4 level, indicating that IL-10 is the relevant factor responsible for the observed protective effects of B10 cells. Moreover, IL-10 production by other cells types, most likely T cells and trophoblasts is not sufficient and cannot compensate the B cell contribution that is missing in µMT mice, at least after LPS challenge.

In the future, the research focus should not only be to target cytokines^43^ but also the cells that are of potential therapeutic interest. It is important not only to study the frequency of the cells but their functionality in big cohorts of patients. It would be very useful to understand whether patients that suffered from infection-associated IUFD, in particular induced by gram-negative infections, had abnormalities in their IL-10 profile or whether B cells from these patients were unable to produce IL-10. In mouse models, we know that it is possible to prevent pre-term birth by applying IL-10^10^. Similarly, rrIL-10 applied exogenously could diminish LPS-induced fetal loss and attenuate growth restriction in rats^44^. Same effects were observed in models of endotoxin-induced fetal loss^45^. Therefore, combined early detection of B cell function and exogenous IL-10 therapy emerge as a promising tool for detecting patients at risk. In the light of our experimental mouse data, we speculate that in the presence of inflammatory signals, a low number of or functionally impaired B10 cells can fracture fetal tolerance and end in fetal loss even in the absence of clinical signs of infection.

Knowing that IL-10 is the main mechanism used by B cells to protect pregnancies from LPS injury in our model, we next aimed to identify some of the pathways activated by B10 cells. It was shown that B10 cells play an important role in the induction and homeostasis of Treg cells^46^ and Treg cells might be crucial in the prevention of LPS-induced pre-term delivery^47^. Bizargity *et al*. have also shown that mice lacking all T and B cells (Rag1^−/−^) are more susceptible to LPS-induced preterm delivery^47^, suggesting a potential importance of B cells to contribute to inflammation-induced pregnancy complications, especially in the absence of Tregs. Co-culturing B10 cells with naïve T cells from gd10 WT and µMT mice induced Treg cells. This was not true for the co-culture of Beff cells with T cells of either mouse group. Thus, it is obvious that the intrinsic absence of B10 cells in µMT mice directly affects Treg cell frequencies *in vivo* and finally impacts on pregnancy outcome. The hypothesis that pregnant mothers are prone to infections because of their expanded Treg population appears as one plausible mechanism but it does obviously not account for the susceptibility of µMT mice as they did not have expanded Treg populations and their fetuses succumbed to LPS dose that are harmless to WT mice. The fact that the transfer with B10 cells or treatment with IL-10 restored the Treg pool and prevented IUFD rather suggests that it is the disturbance of the finely tuned immune balance what interferes with the normal development of pregnancy.

Co-culturing Beff cells with naïve T cells from WT and µMT mice induced IL-6 production. It was shown that upon infection, placentas react with an increased secretion of inflammatory molecules such as IL-6 for pathogen clearance^48^. IL-6 can induce IL-17 producing Th17 cells that can in turn exacerbate the inflammatory response that leads to pregnancy failure^49^ or low birth weight^50^, but also supports the anti-microbial defense. In a model of collagen-induced arthritis it was described that WT CD4+ T cells secrete more IL-17A when cultured with IL-10 deficient B cells^40^. This goes in line with our observation of increased IL-17A production by gd10 WT naïve T cells upon co-culturing with Beff cells, but not in µMT T cells even though IL-6 was not affected. Moreover, naïve T cells differentiated into Th17 cells upon co-culturing with Treg cells in the presence of LPS^51^. Th17 cells were shown to execute effective B cell helping functions including proliferation, differentiation and antibody production^52^. This interaction might also contribute to the poor outcome of µMT mice after LPS-induced intrauterine infection.

Depending on the intensity of the infection and how the pregnant mother reacts to it, the disrupted fetal tolerance can either cause fetal death by allowing fetal invasion by the pathogen or tolerance can be quickly restored by eradicating the pathogen. In our model, it is clear that in the absence of B cells, low dose LPS leads to the break of tolerance and death of the fetus. As the dose chosen for this work was compatible with pregnancy in B cell competent mice, the negative pregnancy outcome depends on the missing B cells. If IL-10 producing B cells were given to the pregnant female before LPS challenge, tolerance could be restored and most of the fetuses survived. When Beff cells that do not secrete IL-10 were delivered instead, the protective effect was minimal and maybe due to some residual cells that could still produce IL-10. No protective effect at all could be observed at all when transferring B cells from IL-10 deficient animals. It is tempting to speculate that, *in viv*o, B sufficient, normal pregnant mice are able to react against a pathogen by boosting the inflammatory cells that kill the pathogens while at the same time stimulating the expansion of B10 cells and/or fostering their suppressive capacity to both limit the infection and protect the fetuses.

We conclude that IL-10 secreting B cells are important for a normal healthy pregnancy in particular for the protection of fetuses from the products of gram- negative bacteria. The involved mechanisms include the modulation of cytokines and upregulation of Treg cells to counteract inflammation.

## Methods

### Animals and mouse model

8–12 weeks old C57BL/6 and BALB/c micr were purchased from Janvier (Le Genest-Saint-Isle, France). B6.129S2-*Ighm*^*tm1Cgn*^/J B cell deficient µMT mice and B6.129P2-*Il10*^*tm1Cgn*^/J IL-10-deficient mice were purchased from Jackson laboratories (Bar Harbor, Maine, USA) and bred at our facilities. Animal experiments comply with the ARRIVE guidelines and were carried out according to institutional guidelines after Ministerial approval and in conformity with the European Communities Council Directive (EU Directive 2010/63/EU for animal experiments; approval number: 42502-2-1332 Uni MD).

Virgin µMT or WT females were mated with BALB/c males. The presence of a vaginal plug marked gd0. For titration of LPS concentration needed to induce IUFD in WT or µMT mice, females were challenged with 0.5 µg/ml LPS, 2 µg/ml LPS, 3 µg/ml LPS or 4 µg/ml LPS at gd10. For all following experiments, 200 µl LPS (3 µg/ml) were i.p. injected on gd10. Control mice received 200 µl PBS. Females were sacrificed at gd11 and the abortion rate was determined.

### High frequency ultrasound

Pregnancy was followed up using non-invasive high-resolution imaging (Vevo® 2100, VisualSonics, Amsterdam, Netherlands) as previously described^53^.

### Isolation of B cells; generation and transfer of B10 cells; IL-10 administration

The Regulatory B cell isolation kit (Miltenyi Biotec, Bergisch Gladbach, Germany) was used to recover non-IL-10 secreting B effector cells (Beff) and IL-10 secreting regulatory B cells (Breg) from spleens of non-pregnant C57BL/6 female mice according to the manufacturer’s protocol. Briefly, B cells were pre-enriched by depletion of non-B cells and stimulated with LPS *E*. *coli* serotype 0111 (10 µg/ml) for 20 h at 37 °C and 5% CO_2_ with the addition of PMA (50 ng/ml) and ionomycin (500 ng/ml) for the last 5 h. Cells were harvested and incubated with the Regulatory B cell Catch Reagent at 37 °C for 45 min. Afterwards, Breg and Beff cells were enriched. B cells from IL-10^−/−^ mice were isolated using the B cell isolation kit (Miltenyi Biotec). 5 × 10^5^ Beff or Breg cells were diluted in 200 μl PBS and injected i.v. into µMT females at gd8 or gd10 (−2d or −2h). B cells from IL-10^−/−^ mice were applied i.v. at gd8 (−2d). 2.5 µg rmIL-10 (R&D System, Germany) was injected i.p. in µMT mice at gd8. 2d or 2 h later, LPS was applied (Fig. 4a; Suppl. Fig. 3a).

### Sample collection and histology

24 h after LPS administration, blood was obtained and centrifuged at 7000 rpm for 10 min at RT. Serum was stored at −80 °C. One placenta per mouse was cultured in 500 µl RPMI1640 + 10%FCS + 1%penicillin/streptomycin. After 24 h, supernatants were collected and stored at −80 °C. One implantation site and one placenta per female were collected 24 h after LPS challenge on gd11 for paraffin embedding. Implantation sites were previously fixed in 4% (w/v) PFA with 0.1 M sucrose (pH 7.4) for 6 h; placentas were fixed in 96% ethanol. Samples were cut longitudinally into 5 μm sections and stained with H&E.

### Cell staining and flow cytometry

Single-cell suspensions from spleen, peritoneal lavage, uterus, blood, inguinal (ILN) and para-aortic lymph nodes (PLN) were obtained and stained for cell surface markers for 30 min at 4 °C. The following anti-mouse fluorescently labeled antibodies were used: CD19 (clone 1D3) and CD4 (clone RM4-4; both from BD Biosciences, Germany), CD25 (clone 3C7), IgM (clone RMM-1), IgD (clone 11-26 c.2a) and B220 (clone RA3-6B2; all from Biolegend, San Diego, CA, USA). For detection of the intracellular expression of Foxp3, cell suspensions were fixed ON using Fix and Perm (ebioscience, Germany) and stained with anti-mouse Foxp3 (clone FJK-16s; ebioscience) for 30 min at 4 °C. Measurements were performed with a FACSCalibur and analyzed with CellQuestPro software (BD Biosciences).

### Cytokine detection in sera and supernatants

Cytokines were quantified by the cytometric bead array (CBA) mouse Th1/Th2/Th17 Cytokine Kit from BD Biosciences, following supplier’s recommendation.

### B cell:T cell co-culture

Beff and B10 cells were obtained as mentioned above. CD4+CD25− naïve T cells from gd10 WT and µMT mice were isolated using a Treg isolation kit (Miltenyi Biotec,). 1 × 10^5^ T cells were cultured with 1 × 10^5^ Beff or B10 cells in anti-CD3 (10 µg/ml; clone 145-2C11) coated plates with soluble anti-CD28 (1 µg/ml; clone 37,51; both BD Biosciences) for 72 h at 37 °C and 5% CO_2._ Supernatants were harvested and cells were stained as described above.

### Data analysis and statistics

Statistical analysis was performed using GraphPad Prism 5.0 software. Normality of distribution was determined by Shapiro-Wilk test. Data were analyzed by Kruskal-Wallis test and Mann-Whitney-U test, for the co-culture repeated measures two-way ANOVA followed by Bonferroni correction was used. Number of animals or samples as well as the statistical test employed for each particular experiment is indicated in the Figure Legend.

## Supplementary information

Supplementary Figures 1-4
