# Supplementary material for: IL-10 producing B cells rescue mouse fetuses from inflammation-driven fetal death and are able to modulate T cell immune responses
Source: Sci Rep. 2019 Jun 27;9:9335. doi: 10.1038/s41598-019-45860-2 (PMC6597542; doi:10.1038/s41598-019-45860-2)
Supplement: Supplementary file 1 — Supplementary Figures 1-4 [file 41598_2019_45860_MOESM1_ESM.pdf]

Supplementary Information

IL-10 producing B cells rescue mouse fetuses from inflammation-driven fetal death and are able to modulate T cell immune responses

Mandy Busse, PhD[1], Kim-Norina Jutta Campe[1], Desiree Nowak[1], Anne Schumacher, PhD[1], Susanne Plenagl[1], Stefanie Langwisch[1], Gisa Tiegs, PhD[2], Annegret Reinhold, PhD[3], Ana Claudia Zenclussen, PhD[1*]

1: Experimental Obstetrics and Gynecology, Medical Faculty, Otto-von-Guericke University, Magdeburg, Germany.
2: Institute of Experimental Immunology and Hepatology, University Medical Center, Hamburg-Eppendorf, Hamburg, Germany.
3: Institute for Molecular and Clinical Immunology, Medical Faculty, Otto-von-Guericke University, Magdeburg, Germany.

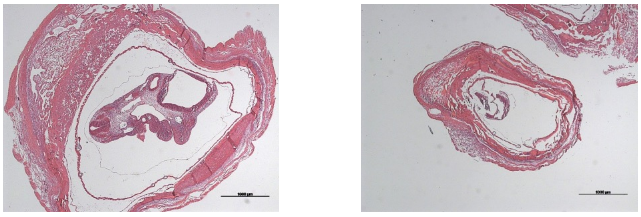

WT gd10 **µMT gd10**

Supplementary Figure 1

Supplementary Figure 1:
Here, representative pictures from H&E staining of WIS, obtained at gestation day (gd10) from WT and µMT mice, are shown. Bars show magnification. The pictures were obtained using 10x ocular and 2,5x objective lens.

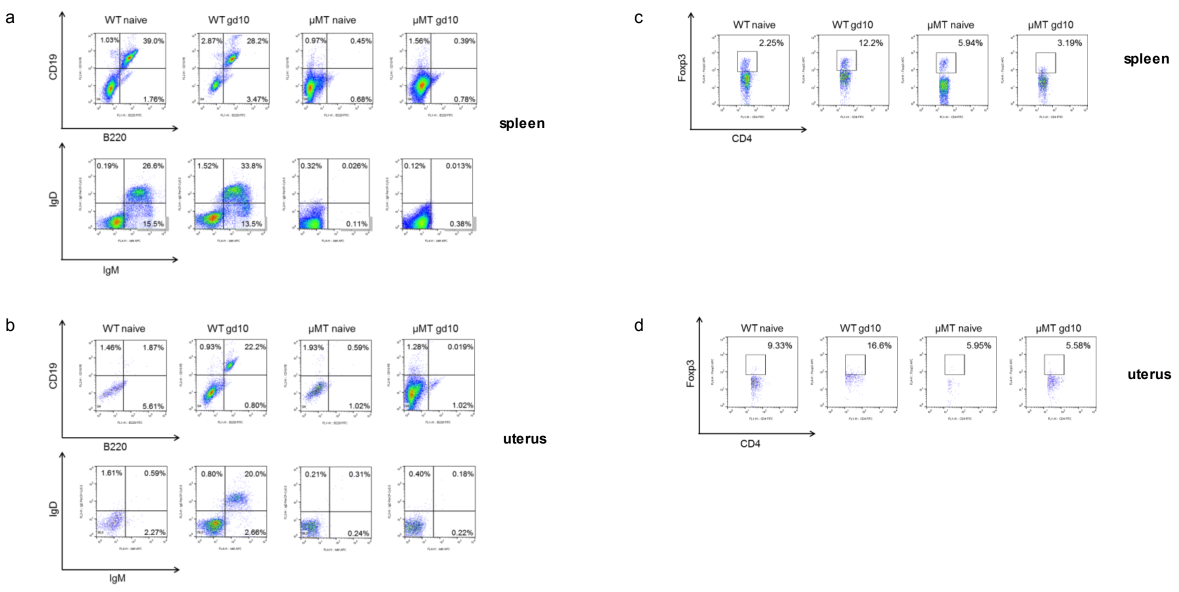

Supplementary Figure 2

Supplementary Figure 2:

Representative dot plots for splenic (a) and uterine (b) B cell populations are depicted for wild type (WT) controls and µMT mice in non-mated (naïve) state as well as at gd10. The analysis was based on the CD19/B220 staining (upper panels) and IgD/IgM staining. Representative dot plots for CD4+Foxp3+ Treg populations for spleen (c) and uterus (d) of naïve and gd10 pregnant WT control and µMT mice. After gating on lymphocytes, the CD4+ T cells were pre-gated and from this population the frequency of Foxp3-expressing cells was calculated. In indicated numbers represent the number of Treg cells within the CD4+ cell population.

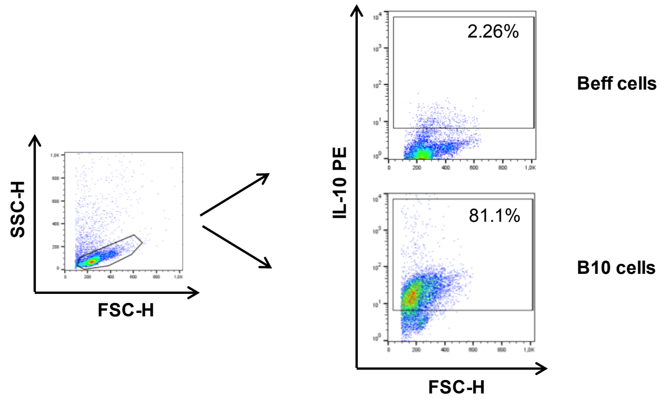

Supplementary Figure 3

Supplementary Figure 3:
Suppl. Figure 3 shows IL-10 negative cells (called B effector cells, Beff) and IL-10 positive cells (B10 cells) as they were obtained from donor mice right after adoptive transfer. After depletion of non-B cells, B cells were stimulated with LPS for 20h, followed by the addition of PMA and ionomycin for the last 5h. Since IL-10 catching reagent was Phycoerythrin (PE) labeled, the purity of IL-10 (PE) negative Beff cells and IL-10 (PE) positive B10 cells was determined following isolation, directly before i.v. injection into gd8 pregnant µMT mice.

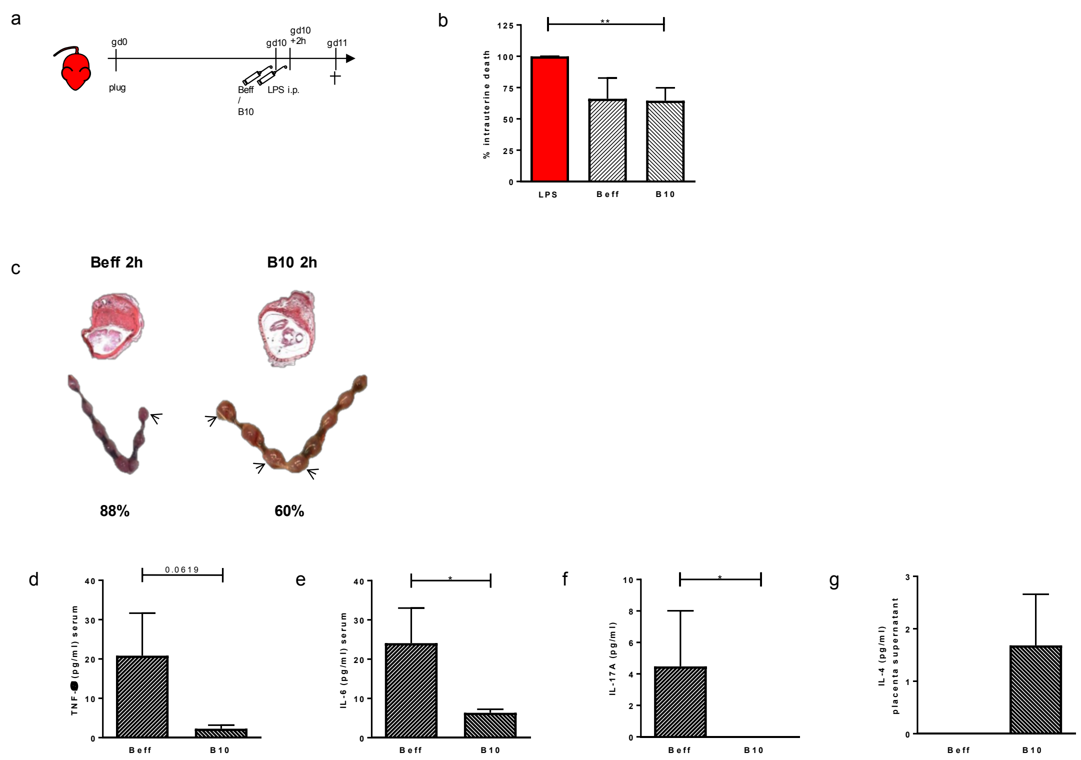

Supplementary Figure 4

Supplementary Figure 4:

Suppl. Figure 4a shows the experimental design of the adoptive cell transfer experiment. µMT mice were supplemented i.v. with Beff or B10 cells obtained from WT mice 2h before LPS challenge at gd10. 24h after LPS, mice were sacrificed. In b), the IUFD of all groups is shown. Injection of B10 cells 2h before LPS improved significantly fetal survival. In c), representative pictures of H/E stained implantations (upper part) as well as photos from uteri of µMT mice supplemented with Beff or B10 cells at gd10, followed by LPS injection 2h later, and killing of the dams 24h later, are presented (lower part). The % of IUFD corresponds to the examples in (d), arrows mark living fetuses. (e-g) cytokine levels in serum of treated animals. B10 cell injection reduced TNF-α (d), IL-6 (e) and IL-17 (f) compared to Beff-treated dams. Placenta supernatants from B10-treated dams had increased IL-4 (g) compared to Beff-treated mice. In (g) no statistic test was possible because all samples from the Beff group were under the detection limit. Kruskal-Wallis test and Mann-Whitney-U test were used to analyze the data. N=5-8 mice/group; $*p<0.05$ $**p<0.005$.
